# Overcommitment to work as a mediator of the association between effort-reward imbalance and insomnia among shift working nurses

**DOI:** 10.1192/j.eurpsy.2021.2160

**Published:** 2021-08-13

**Authors:** K. Gustavsson, A. Wichniak

**Affiliations:** 1 Department Of Clinical Neurophysiology, Sleep Medicine Center, Institute of Psychiatry and Neurology, Warszawa, Poland; 2 Third Department Of Psychiatry, Institute of Psychiatry and Neurology, Warszawa, Poland

**Keywords:** occupational stress, Insomnia, overcommitment, Shift Work

## Abstract

**Introduction:**

Today, approximately one fifth of employees in the European Union works in the shift system. Insomnia is one of the most common consequences of occupational stress and shift work. Identifying factors contributing to poor sleep quality among shift workers, especially in healthcare professions, is important because insomnia increases the risks for numerous health disorders and impacts work ability.

**Objectives:**

The aim of this research was to investigate to what extend does an inability to withdraw from work influences the link between occupational stress and insomnia among shift workers. We operationalized occupational stress within the Effort-Reward Imbalance Model (ERI). An imbalance between individual effort and reward obtained at work leads to experiencing a stressful work environment.

**Methods:**

153 shift working female nurses completed a short questionnaire about work schedule, the Effort-Reward Imbalance questionnaire with the Overcommitment (OC) scale and the Insomnia Severity Index (ISI). To estimate the mediating effect of OC on the association between ERI and ISI, we conducted a mediation analysis using PROCESS v3.4 macro in SPSS.

**Results:**

The model including ERI and OC accounted for 12.25% of the variance in ISI scores. ERI significantly predicted OC and OC significantly predicted insomnia. The direct effects of ERI on ISI remained significant after controlling for the effects of overcommitment. 35.07% of the effect of ERI on ISI was through OC.

**Conclusions:**

Ability to detach from work-related thoughts during leisure time is crucial for successful recovery from occupational stress. The research is supported by a grant no. 2019/33/N/HS6/02572 from the National Science Center in Poland.

**Disclosure:**

No significant relationships.

